# Ultraviolet A1 Phototherapy for Fibrosing Conditions

**DOI:** 10.3389/fmed.2018.00237

**Published:** 2018-08-27

**Authors:** Thilo Gambichler, Lutz Schmitz

**Affiliations:** Department of Dermatology, Ruhr-University Bochum, Bochum, Germany

**Keywords:** UV-A1, ultraviolet A1, UVA1, irradiation, sclerosis, fibrosis, phototherapy

## Abstract

In this article we describe efficacy and safety aspects of ultraviolet A1 (UV-A1) phototherapy in fibrosing conditions. UV-A1 is a specific phototherapeutic modality that is defined by a selective spectral range (340–400 nm). UV-A1 includes distinct modes of action qualifying this method for therapy of a variety of conditions, in particular fibrosing skin diseases. Concerning efficacy of UV-A1 phototherapy in fibrosing conditions, the best evidence obtained from randomized controlled trials exists for localized scleroderma. Moreover, fibrosing disorders such as lichen sclerosus and graft-vs.-host disease can be treated successfully by means of UV- A1. Regarding the optimal dosage regimen medium-dose UV-A1 seems to be linked to the best benefit/risk ratio. Possible acute adverse events of UV-A1 phototherapy include erythema and provocation of photodermatoses. Skin ageing and skin cancer formation belong to the chronic adverse events that may occur after long-term UV-A1 phototherapy.

## Introduction

In order to reduce adverse effects such as erythema UV-A1 (340–400 nm) light sources were previously developed by eliminating the UV-A2 wavelengths (320–340 nm) which range to the UV-B (280–320 nm) spectrum (1, 2). Compared to UV-B and UV-A2, UV-A1 is thus less erythematogenic and does penetrate deeper into the skin (3). UV-A1 is a beneficial phototherapeutic modality for the treatment of disorders including eczema, urticaria pigmentosa, cutaneous T cell lymphoma, and in particular, fibrosing skin diseases (4–14). The present review focuses only on the fibrosing skin diseases and, although UVA1 may be beneficial in other conditions, they are not the focus of this review. We will also summarize the evidence in table format for each of the diseases discussed in the following review.

### UV-A1 light sources and regimens

#### UV-A1 devices

Fluorescent bulbs (i.e., TL10R 100W, Philips, Eindhoven, Netherlands) and high-output metal halide lamps (i.e., Sellamed 4,000W, Sellas Medizinische Geräte GmbH, Ennepetal, Germany) belong to the commercially available UV-A1 sources. For practical reasons, fluorescent lamp cubicles are rather used for low to medium-dose UV-A1 phototherapy. By contrast, high-output metal halide lamps can also be used for high-dose UV-A1 since they deliver doses up to 130 J/cm^2^ in acceptable time per treatment session. UV-A1 light sources designed for phototherapy have to fulfill some technical requirements. Hence, the amount of wavelengths smaller than 340 nm must be smaller than five percent of the total erythema-effective fluence. Furthermore, wavelengths smaller 320 nm as well as infrared should also be widely filtered out. Thus, irradiance of wavelengths between 800 nm and 1 mm must not be greater than five percent of the total fluence (15). Fluorescent lamp whole- body devices are relatively inexpensive but have considerably lower spectral output as compared to metal halide devices (15). Nevertheless, duration of irradiation using high-output UV-A1 beds may also be long as the patient must usually treat subsequently to two body sides (15). Using UV-A1 metal halide lamps exposure times of thirty to sixty minutes per session are not uncommon, of course depending on fluence, indication and dosage regimen (14, 16).

#### Dosage regimens

In order to be consistent with previous publications that are discussed in the present review we use the dosage categories as follows: low-dose UV-A1 (10–20 J/cm^2^), medium-dose UV-A1 (>20–70 J/cm^2^), and high-dose UV-A1 (>70–130 J/cm^2^). Before starting UV-A1 phototherapy the medical history (i.e., photo skin-type, sun sensitivity, skin cancer) of the patient has to be checked also including the use of photo-allergic medications and immune-mediated photodermatoses. Importantly, immunosuppressants, including azathioprine, must not be combined with UV-A1 (17).

Given that there may be considerable variability in individual susceptibility to UVA1 erythema, undertaking an MED prior to starting treatment is preferred where feasible. If this proves not to be the case then a fixed start dose, for example, 20 J/cm^2^ would usually be a safe approach, but there would then be the concern of potential under-treatment if running at doses that are well below the erythemal threshold (18, 19). UV-A1-MED data of two recent studies indicate that 20 J/cm^2^ do usually not lead to erythema (20–22). Regular UV-A1 dosimetry is highly recommendable. The irradiance of the light sources should be assessed at different test sites whereby the mean value of all measurements defines the irradiance used for dose calculations (23).

### Localized scleroderma

High-dose UV-A1 therapy of localized scleroderma (LoS) was first reported by German researchers in 1997 (24). Stege et al. compared 10 patients receiving high-dose UV-A1 therapy with seven patients who were exposed to low- dose UV-A1 therapy. Stege et al. showed that UV-A1 significantly increased skin elasticity and decreased thickness and stiffness of the skin–these effects were particularly seen following high-dose UV-A1 (24). The latter findings are supported by *in vitro* analyses showing UV-A1 to reduce cell proliferation and dose-dependently decrease of collagen and hydroxyproline levels.

Moreover, a mouse model of scleroderma showed for high-dose UV-A1 a marked therapeutic effect on scleroderma. An improvement of dermal sclerosis and softened skin tissue could be observed (25). These results are in line with another mouse model study by Karpec and colleagues who investigated in scleroderma patients the impact of high-dose UV-A1 on dermal sclerosis. They could demonstrate that a total dose of 1,200 J/cm^2^ does obviously not only prevent worsening of dermal fibrosis but also leads to a decrease of fibrotic skin changes (26). A further study by this working group showed in an animal model employing bleomycin- induced scleroderma that UV-A1 (cumulative doses: 1,200 J/cm^2^ and 600 J/cm^2^) is effective as well safe in the management of scleroderma (27).

By contrast, there is a wealth of data confirming the efficacy of low-dose UV-A1 therapy. Kerscher et al. (28) reported for the first time on a successful low-dose UV-A1 treatment in LoS patients (*n* = 10). Later, they conducted a study including 20 LoS patients who were treated with low-dose UV-A1 over 12 weeks. UV-A1 resulted in remarkable clinical improvement in 80% of the patients (29). However, patients (*n* = 2) with subcutaneous LoS did not respond to treatment. In a small study performed by Gruss and co-workers, the results mentioned above were supported as well (29). Moreover, LoS patients were treated three times per week using UV-A1 phototherapy (30 J/cm^2^, treatment duration 10 weeks) (30). In all patients, softening of skin lesions was reported by the authors (30).

de Rie et al. reported on a controlled medium-dose UV-A1 trial including eight patients suffering from LoS (31). UV-A1 was given four times weekly over three months resulting in a decrease of skin fibrosis (cumulative dose: of 2,304 J/cm^2^ UV-A1). We previously performed a comparative trial investigating low- dose UV-A1 (20 J/cm2), medium-dose UV-A1 (50 J/cm2), and narrowband UV-B for patients with LoS (32). Sixty-four patients suffering from LoS were treated in a randomized controlled trial including three treatment arms (15). Severity of LoS was evaluated using a simple clinical score. Phototherapy was performed five times weekly over two months. Kreuter (32) observed a significant improvement of LoS in all patients who completed the study which was shown by a decrease of clinical symptoms in all study arms assessed (15, 32). However, medium-dose UV-A1 was significantly more effective than narrowband UV-B (32). While low-dose and medium-dose UV-A1 were equally beneficial, substantial differences between low-dose UV-A1 and narrowband UV-B and medium-dose UV-A1 could not be observed.

Sator et al. (33) treated three clinically comparable LoS plaques in sixteen patients using 20 J/cm^2^ UV-A1, 70 J/cm^2^ UV-A1, or non-irradiation (32). Thirty therapy sessions were applied in total. Sator et al. (33) assessed thickness of the skin using high-frequency sonography and clinical score. Sonography revealed a significantly greater decrease of skin thickness for medium-dose UV-A1 when compared to low-dose regimen. By contrast, clinical scoring of fibrotic lesions irradiated also decreased markedly but did not show a clinically meaningful difference between medium-dose and low-dose UV-A1 (32). Together, the authors found that medium-dose UV-A1 for LoS resulted in more favorable long-term results when compared to low-dose UV-A1 as confirmed by sonographic assessments. High-frequency sonography is likely a more sensitive tool for the assessment of UV-A1-induced skin changes in LoS patients (33).

A recent cohort study by Vasquez and colleagues investigated recurrence risk of morphea after successful UV-A1 therapy–they observed the duration of LoS prior to therapy as the only associated variable. There was no difference in recurrence risk between different subtypes of morphea, skin types, adults and children, and medium to high dose regimens. Thus, the authors conclude that treatment doses in the medium- and high-dose UV-A1 range are adequate regarding the frequency of recurrence (34). Su et al. (35) treated 35 LoS patients with medium-dose UV-A1 (30 J/cm^2^). Medium-dose UV-A1 therapy improved fibrotic lesions in all patients. A substantial treatment success was found in 29 of 35 patients. Ultrasound measurements demonstrated that the thickness of skin significantly decreased after medium-dose UV-A1. There were no detectable treatment related adverse events.

Moreover, Andres et al. (36) demonstrated in LoS patients a favorable short-and long-term effect through medium-dose UV-A1 therapy, including diminishment of fibrotic lesions, improvement of skin elasticity, and decrease of skin thickness. Furthermore, Pereira et al. (37) conducted a retrospective evaluation of LoS patients who had underwent low-dose UV-A1 (average dose: 31 J/cm^2^) phototherapy (32). They treated 18 patients with LoS showing a substantial improvement in more than three-fourth of patients and a modest improvement in 12% of patients (37). Moreover, Gruss et al. (38) reported on disabling pansclerotic morphea of childhood who was successfully treated with low-dose UV-A1 (cumulative dose: 640 J/cm^2^ UV-A1) four times weekly over two months resulting in substantial reduction of skin fibrosis.

Together, medium UV-A1, based on the evidence base would be considered as the phototherapeutic treatment of choice for patients with LoS (Table 1). However, it is worth emphasizing that there is no head-to-head comparison between UV-A1 and psoralen plus UV-A (PUVA) for scleroderma, and this would be an important study with regards to establishing the place of UVA1 in the phototherapeutic approaches of scleroderma, as at present we do not know whether UVA1 is equivalent, inferior or superior to PUVA.

**Table 1:** UV-A1 treatment for fibrosing conditions—levels of evidence as proposed by the American College of Cardiology and the American Heart Association (39).

**Levels of evidence**	**Indications/protocol**
**Level A**	
Data derived from multiple randomized clinical trials or Meta-analyses	Localized scleroderma ^§^Medium-dose 60 J/cm^2^ 3–5 times weekly total of 40 sessions
**Level B**	
Data derived from a single randomized trial, non-randomized studies, prospective case studies	Lichen sclerosus ^§^Medium-dose 50 J/cm^2^ 5 times per week total of 40 sessions
**Level C**	
Only consensus opinion of experts, retrospective case studies, case reports, or standard-of-care	Systemic sclerosis^*^ Nephrogenic systemic fibrosis GvHD

* conflicting data,

§ *medium-dose UVA1 >20–70 J/cm*.

### Systemic sclerosis

von Kobyletzki et al. (40) reported on eight patients suffering from systemic sclerosis (SSc) whose acrosclerosis was treated with low-dose UV-A1. They used 30 J/cm^2^ UV-A1 four times per week over two months and thereafter three times weekly over a six week period (50 treatment sessions in total, cumulative dose: 1,500 J/cm^2^) (40). Morita et al. (41) also observed UV-A1-induced softening of skin fibrosis (cumulative dose: 510 to 1,740 J/cm^2^) in four patients with SSc. In another paper they also found UV-A1-induced decrease of dermal decorin expression in SSc patients (41, 42). In an open non-randomized study we previously treated 18 patients with acrosclerosis and underlying SSc. Applying the UV-A1 regimen described by von Kobyletzki et al. (40), Kreuter et al. (43) observed skin softening, enhancement of skin distension, decrease of thickness of skin, and increase of cutaneous collagenase activity in 16 of 18 patients (32).

Pereira et al. (37) reported three SSc patients who were treated with medium-dose UV-A1. In two patients, acrosclerosis improved significantly (37). Moreover, Rose et al. reported on eight SSc patients (diffuse type, *n* = 5; limited type, *n* = 3) who showed skin fibrosis predominantly on acral and proximal extremity sites. The patients were treated using UV-A1 (30–40 J/cm^2^) 3 times per week. Skin fibrosis improved as indicated by a decrease of the modified Rodnan skin score (32). Hence, this study also demonstrated that UV-A1 treatment is effective in SSc patients, particularly for acrosclerosis (44). In contrast, Durand et al. (45) reported a randomized observer-blinded half-side controlled trial on UV-A1 treatment of acrosclerosis. They used low-dose UV-A1 (40 J/cm^2^) three times per week (14 weeks treatment period). Although a marked improvement of the clinical scores was observed, no difference could be detected regarding the clinical outcome of irradiated and non-irradiated extremities (32).

In contrast to the aforementioned results, the data of Durand et al. (45), which was based on a controlled investigation, suggest that UV-A1 therapy is ineffective in acrosclerosis (45). Otherwise one must consider a systemic UV-A1 effect that could explain the results of Durand et al. (45). Moreover, Tewari et al. reported medium-dose UV-A1-induced reduction of microstomia in a SSc patient (46). Jacobe et al. (9) effectively treated 34 SSc patients. On the basis of their data, medium- to high-dose UV-A1 therapy seems to be similarly effective independently of patients photo-skin types. Nevertheless, outcome measures were not reported in detail (9). In another study on 16 SSc patients, a statistically significant dose-response association was found between low-, medium-, and high-dose treatment regimens (47). Notably, Comte et al. reported UV-A1- induced improvement of Raynaud's phenomenon observed in over 80% of patients (*n* = 11) with autoimmune disorders including SS (48).

In contrast to the well-documented evidence of beneficial UV-A1 efficacy in LoS the data for SSc are pretty contradictory and of much poorer quality. Hence, UV-A1 should not be considered a first-line treatment modality for SSc patients.

### Lichen sclerosus

In a prospective non-controlled study, we treated ten patients suffering from extragenital lichen sclerosus (LiS) with low-dose UV-A1 (20 J/cm^2^) therapy 4 times weekly (32). After low-dose UV-A1 therapy a remarkable decrease of the clinical score and normalization of skin texture was observed as also confirmed by sonography. The patients noticed substantial skin softening and repigmentation in pre-existing lesions. It was suggested that similar to therapy outcomes in LoS, low- dose UV-A1 therapy seems to be a beneficial and well-tolerated therapy modality for extragenital LiS (32). Rombold et al. (11) also observed beneficial outcome for LiS patients managed with medium-dose UV-A1 (cumulative dose: 1,018 ± 575.3 J/cm^2^).

Beattie et al. (49) evaluated the efficacy of UV-A1 in genital LiS. Seven females were exposed to UV-A1 (low- to high-dose protocol according to MED). Five patients responded to treatment, three patients showed modest clinical improvement, and two experienced only slight therapy success. Of the five responders, one had disease relapse within three months and another after one year. The latter patients were re-treated by means of UV-A1 therapy – one had minimal improvement, the other had moderate treatment success. In the other responders, the condition substantially improved and was controllable using topical glucocorticosteroids. The authors suggested that UV-A1 is potentially an effective treatment approach for genital LiS, particularly considering that this disease is frequently poorly manageable (49).

Data of a randomized controlled trial performed in our department comparing the efficacy of high-potent topical glucocorticosteroids (clobetasol propionate 0.05%) with UV-A1 therapy (50 J/cm^2^, 4 times per week over 12 weeks) in the management of 30 patients with genital LiS showed a significant improvement of symptoms. Nevertheless, the current gold standard, say high-potent glucocorticosteroids, was superior to UV-A1, particularly with respect to practical considerations, reduction of pruritus, and quality of life improvement. However, we suggested to consider UV-A1 phototherapy as potential second-line treatment for VLiS (50). Moreover, our study group investigated epigenetic changes in 10 patients with LiS before and after a medium-dose UV-A1 (up to 50 J/cm^2^, 4 times weekly for 3 month) treatment compared to healthy controls. It could be shown that UV-A1 phototherapy may cause a normalization of 5-hydroxymethylcytosine levels–epigenetic factors may also contribute to LiS pathophysiology (15, 51).

Conclusively, based on data derived from a single randomized trial, non-randomized studies, and prospective case studies UV-A1 appears to be a treatment option for genital and extragenital forms of LiS.

### Graft-vs.-host disease

Previously, Grundmann-Kollmann et al. (52) reported a patient suffering from chronic sclerodermic graft-vs.-host disease (GvHD) who was refractory to conventional therapies (32). In the combination with oral mycophenolate mofetil low-dose UV-A1 (20 J/cm^2^) four times weekly was beneficial (cumulative dose: 480 J/cm^2^ UV-A1). Furthermore, Stander et al. (53) studied five GvHD patients receiving 50 J/cm^2^ UV-A1 (5 times per week) over eight weeks followed by subsequent diminishment of UV-A1 doses toward 3 times weekly (32). Notably, one patient was irradiated using a fix dose of 20 J/cm^2^ UV-A1 combined with immunosuppressants and extracorporeal photopheresis (ECP). In all patients, treatment resulted in skin softening of pre-existing lesions (53). Calzavara-Pinton et al. (54) treated five patients with sclerodermoid GvHD (localized: 4; generalized: 1) with medium-dose UV-A1 (50 J/cm^2^) therapy three times weekly. Therapy was successful with complete responses observed in three patients and partial responses in two (54).

In contrast, a study of 25 GvHD patients by Connolly et al. found clinical improvement in patients who received high-dose UV-A1 phototherapy (47). In a small trial, 7 patients were exposed to UV-A1 as primary treatment for acute cutaneous GvHD. In 5 patients, a complete response was noticed, in 2 patients were non-responders and requiring systemic steroids (32). In 2010, Schlaak et al. (55) studied 70 patients suffering from acute cutaneous GvHD. Following a median therapy period of 10 months, the authors achieved complete and partial responses in 70% and 24.3% of patients, respectively. Following a median follow-up of 18 (range 10–60) months, non-melanoma skin cancer occurred in three patients. The authors concluded that UV-A1 therapy can be a beneficial therapy for acute GvHD affecting the skin (32). Avoiding chronic use of systemic glucocorticosteroids and/or allowing a faster tapering of immunosuppressants in a substantial number of patients, UV-A1 appears to be an interesting therapy option for GvHD (55). Moreover, Ziemer et al. treated two children with chronic cutaneous GvHD who improved after UV- A1 therapy with regard to cutaneous lesions, joint mobility, and quality of life (32).

The benefit of UV-A1 for GvHD patients has only been documented in small retrospective case series and case reports making it difficult to give a definitive recommendation for this photherapeutic modality in GvHD. Moreover, there are no comparison studies with UV-A1 and ECP–a frequently recommended photochemotherapeutic option for GvHD patients.

### Nephrogenic systemic fibrosis

Tran et al. (56) recently treated nephrogenic systemic fibrosis (NSF) with UV-A1 phototherapy. All patients (*n* = 4) received hemodialysis before, during, and after high-dose UV-A1 (32). All patients noticed softening of their skin, and two patients experienced increase of mobility of the limbs. The therapeutic was significant in all cases, even though none patient complete clearance of fibrosis could be achieved. Hence, UV-A1 represents a feasible therapy modality for NSF, in particular in cases in which kidney transplantation is no option or in delay (56). Interestingly, UV-A1 does not only improve clinically NSF but also induce procollagen synthesis and reduce profibrotic cytokine and growth factor expression (32). Using a medium-dose regimen, however, we could not observe beneficial effects after UV-A1 therapy in patients (*n* = 3) with NSF (32). These results are supported by an analysis of 17 patients with NSF which found high-dose regimens to be more effective than medium- and low-dose regimens for NSF (47). By the way, Gazi et al. (57) performed a survey, and found that an reduction of 3 to 7.5 points of the modified Rodnan skin score does reflect a clinically meaningful treatment outcome (32).

In conclusion, UV-A1 may work in NSF; however, this statement is only based on a few case series and retrospective observations.

### Miscellaneous

Moreover, positive results following UV-A1 phototherapy of fibrosing conditions have been documented in case reports on patients with scleromyxedema, scleredema adultorum Buschke, and pansclerotic porphyria tarda. Variable data have been reported for UV-A1 therapy of keloids and eosinophilic fasciitis (5, 7, 11, 58–63).

### Mechanisms of action, limitations, and adverse events

#### Photo-skin type status

It is still controversially discussed whether patients with photo-skin type > III respond worse to UV-A1 therapy (7, 45, 64). Wang et al. (64) demonstrated that a single UV-A1 dose can markedly reduce procollagen mRNA gene expression and substantially enhance matrix metalloproteinase 1 and 3 gene expression in controls (15). Their results showed that such anti-fibrotic effects likely decrease after repeated UV-A1 irradiation sessions (15). By contrast, skin darkening usually depends on dosage (15). Stronger pigmentation resulted in a decrease of the anti-fibrotic effects of UV-A1 (15). Hence, individuals with dark skin show only marginal or even no decrease of procollagen when compared to individuals with fair skin (15). The aforementioned results could have significant implications on patient stratification for therapy, proposing that patients with fair skin are better candidates for UV-A1 therapy (15). Wang et al. (64) speculated that the aforementioned observation may be the reason for more favorable outcomes reported in previous UV-A1 trials on sclerotic skin diseases predominantly including European Caucasians (15).

Tuchinda et al. (7) reported (*n* = 92) that patients with fair skin likely respond to UV-A1 better than patients with darker skin (15). However, Jacobe et al. (9) performed a study on 101 patients who were treated with UV-A1 treatment. Photo-skin types and total UV-A1 doses were analyzed. The evaluation of therapy outcome was based on clinical parameters such body surface area, fibrosis and subjective symptoms such as itch (15). Interestingly, clinical response to UV-A1 was not dependent on skin complexion in this population assessed.

#### Mode of action aspects

More infrequent types of LoS including linear LoS an deep morphea and severe cases of acrosclerosis and sGVHD frequently affect deeper anatomical structures such as fascias, muscles, and bones (15). Because UV-A1 penetrates into the subcutis only, the aforementioned conditions rather require systemic immunosuppressive treatment such as methotrexate (65). Evidence indicates that UV-A1 phototherapy acts through diminishment of cutaneous T cell infiltrates, down-regulation of pro-inflammatory cytokines, changes in endothelial cell function, and induction of programmed cell death. Nevertheless, the most important mode of action of UV-A1 in fibrotic conditions is the induction of matrix metalloproteinases and inhibition of collagen synthesis (15). Furthermore, UV-A1 exerts changes in fibroblast cytokine production (15) such as transforming growth factor- ß/Smad signaling and interleukin (IL) and IL-6, leading to an upregulation of collagenase activity (15). It was shown *in vitro* that UV-A1 irradiation of cultured fibroblasts obtained from LoS patients resulted in increased collagenase gene and protein expression. After UV-A1 irradiation, it was also observed a fast production of interleukin 1 (IL-1) stimulating the release of IL-6 which mediates an upregulation of collagenase synthesis by fibroblasts (14, 65–71).

#### Side effects

The most common acute adverse events of UV-A1 include increased pigmentation, erythema, and itch (5, 10, 12, 14, 72). UV-A1 treatment usually needs long exposure times, resulting in considerable heat, which might be intolerable for patients. Phototoxic reactions may occur, in particular in patients with fair skin (20). Notably, UV-A1 absorbing substances of the skin, such as porphyrins and riboflavins, can cause oxidative stress resulting in phototoxic reactions (73). Beside the aforementioned side effects, Wang et al. (74) investigated the effects following a limited number of low-dose UV-A1 irradiation sessions as usually experienced in daily life. They observed that these UV-A1 exposures potentially promoted photoaging by affecting breakdown, rather than synthesis, of collagen. In fair skinned individuals, increasing skin pigmentation due to low-dose UV-A1 did not prevent collagenolytic alterations usually induced by UV-A1. They concluded that sunscreens must block sufficiently UV-A1 wavelengths as well (74). Furthermore, UV-A1 can induce photodermatoses or reactivate herpes flares (16, 75). A recent case study reported a 37-years-old female with a persistent polymorphous light eruption lasting for 5 weeks following UV-A1 phototherapy (76).

Skin cancer and premature skin aging belong to the most important chronic side effects linked to broadband UV-A radiation. UV-A can suppress skin immunity in a bell-shaped dose response (15). Long-wave UV-A corresponding to dose equivalents of 20 min sun exposure contributes to about 75% immunosuppression caused by sun irradiation (15). It was shown that UV-A1 but not UV-A ranging from 320 to 350 nm induces immunosuppression in humans, indicating a significant role for reactive oxygen species (77). Moreover, UV-A induces an energy crisis in cells, can activate alternative complement pathways, and alters the development of memory T cells (15). Skin cancers are associated with p53 and BRM mutations, which can be induced by UV-A1 as well (77–79).

Of importance is also research of Tewari et al. who recently reported a study indicating the induction of DNA dimers at the basal layer and in the upper dermis after UV-A1 exposure (80).

Principally, patients treated with UV-A1 must have regular skin checks and should avoid the use of sunbeds and/or additional sun exposure (15). UV-A1 contraindications may include conditions of UV sensitivity (i.e., xeroderma pigmentosum, porphyrias), use of UV sensitizing substances, history of skin cancers, radiotherapy, and chronic immunosuppresssion (15). For example, azathioprine leads to increased UV-A sensitivity and thus is a well-known photocarcinogen (81).

### Overall conclusion

The best evidence of efficacy for UV-A1 therapy exists in LoS. We consider medium UV-A1 the first-line modality for disseminated forms of this disease, in particular given the fact that there is a lack of effective standard treatments. The latter does also apply to LiS which is closely related to LoS. Hence, UV-A1 represents an attractive treatment option for widespread LiS as well. In the other conditions discussed above UV-A1 may represent an alternative treatment option. About 6 years ago, Kerr et al. (23) considered that UVA1 should only be available through specialist services until we have more evidence. With regard to efficacy of UV-A1 we think that this phototherapeutic option should be widely available in all dermatology centers. However, the price for high-output UV-A1 devices is still very high. Hence, we are afraid that UV-A will predominantly remain a more specialized unit tertiary service.

## Author contributions

Both authors contributed to the literature search, data extraction, interpretation of results, and preparation of the manuscript. Manuscript approval was performed by both authors.

### Conflict of interest statement

The authors declare that the research was conducted in the absence of any commercial or financial relationships that could be construed as a potential conflict of interest.
